# Temporal Consistency Is Currency in Shifts of Transient Visual Attention

**DOI:** 10.1371/journal.pone.0013660

**Published:** 2010-10-28

**Authors:** Árni Kristjánsson, Katrín Ósk Eyjólfsdóttir, Anna Jónsdóttir, Guðmundur Arnkelsson

**Affiliations:** Department of Psychology, University of Iceland, Reykjavík, Iceland; University of Regensburg, Germany

## Abstract

**Background:**

Observers respond more accurately to targets in visual search tasks that share properties with previously presented items, and transient attention can learn featural consistencies on a precue, irrespective of its absolute location.

**Methodology/Principal Findings:**

We investigated whether such attentional benefits also apply to temporal consistencies. Would performance on a precued Vernier acuity discrimination task, followed by a mask, improve if the cue-lead times (CLTs; 50, 100, 150 or 200 ms) remained constant between trials compared to when they changed? The results showed that if CLTs remained constant for a few trials in a row, Vernier acuity performance gradually improved while changes in CLT from one trial to the next led to *worse* than average discrimination performance. The results show that transient attention can quickly adjust to temporal regularities, similarly to spatial and featural regularities. Further experiments show that this form of learning is not under voluntary control.

**Conclusions/Significance:**

The results add to a growing literature showing how consistency in visual presentation improves visual performance, in this case temporal consistency.

## Introduction

We attend to the stimuli of interest and ignore irrelevant ones. This selective attending must be applied to the right stimulus at the right time. Visual characteristics which are stable in space and time are, in general, more easily perceived than otherwise. Consistency of task characteristics facilitates visual and attentional processing. Maljkovic & Nakayama [1] showed how visual search times were speeded when the color of the target was repeated from one trial to the next. This did not simply reflect response facilitation [2]. Similarly, Kristjánsson ([3], see also [4]–[11] and [12] for review) found that when the characteristic features of a Gabor patch were repeated, search was speeded and that priming of different features could be dissociated. Such repetition benefits have even been seen for characteristics of brief precues summoning transient attention [13], [14], usually thought to be an example of automatic processing [15], [16].

Note that such repetition effects are not under explicit conscious control. They can occur without the observers being in any way aware of the repetition pattern (see [17], [18] for review), and can even lead to performance decrements when the learning occurs in violation of instructions given to the observers [1], [14]. Other examples of such benefits from between-trial consistency for visual attention include probability cueing of location [19], [20], where target detection is faster at locations where a target is more likely to appear than in other locations, perceptual learning [21], [22] contextual cueing [23] and statistical learning [24].

### The current goals

Many studies indicate that consistencies in time can benefit visual and attentional performance [25], but this has not been shown to occur on the timescale of transient attention. The literature shows how performance on visual acuity tasks is improved with positional precues of where a target appears among distractors [26]–[28] and there is, indeed, good evidence that observers can effectively attend to instances in time as well as locations in space [29], [30]. The current research was intended to investigate visual performance under temporal consistency versus temporal uncertainty with cues summoning transient attention. In the first experiment, observers performed a cued Vernier discrimination task with brief (50, 100, 150 or 200 ms) precues which indicated where, on an imaginary oval centered on a fixation point at screen center, a target appeared. The critical question was whether Vernier discrimination of an upper line laterally displaced from a lower one would be affected by whether the cue-lead time (CLTs) remained constant for a few trials in a row compared to when the CLTs changed from one trial to the next. Rather than using blocked versus random conditions we pseudo-randomized (see methods section) CLTs within blocks to look at cases where the CLTs remained the same for different numbers of trials in a row versus when they changed between trials (see [1], [14]). The observers performed a large number of experimental trials to ensure enough trial-to-trial combinations of different CLTs to address the experimental question with sufficient power. We estimated discriminability of the Vernier targets as a function of the critical temporal manipulations, by plotting psychometric functions. By measuring changes in slope of the psychometric function we can obtain estimates of how discriminable leftwards versus rightwards displacement are.

In experiment 2 we attempted to disentangle benefits from consistency upon spatial cueing effects from a general non-spatial benefit from temporal consistency, and experiment 3 was conducted to investigate whether explicit knowledge of the CLTs on any given trial can aid performance on a discrimination task involving transient attention.

## Results

### Experiment 1 – The effects of between-trial temporal consistency in precued deployments of transient attention

#### Participants

Four observers participated, ranging in age from 22 to 23. Two of the observers (AJ and KOE) were authors and knew about the purpose of the experiment while the other two participants were naïve. All had normal or corrected-to-normal vision. Each observer participated in 6000 trials and in at least 30 practice trials beforehand, or until they were comfortable with performing the task. Testing was spread out over a number of days, dependent upon the convenience of each observer.

#### Materials

The stimuli were presented on a 100 Hz CRT screen with a spatial resolution of 1024 by 768 pixels, controlled by a 400 MHz Power PC G4 computer. Custom software, programmed in C, utilizing functions from the VisionShell library, was used for stimulus presentation.

#### Stimuli

**Figure 1:**
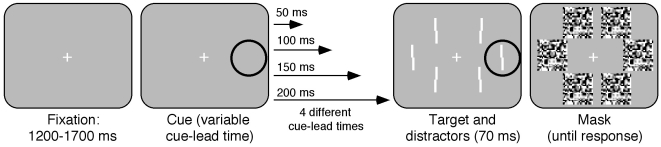
The sequence of events on a single experimental trial in experiment 1. A fixation point was presented for 1200 to 1700 ms (determined randomly for each trial) followed by the cue presented for 50, 100, 150 or 200 ms (see methods section for details upon the probability of a given CLT). Following the presentation of the cue, the Vernier acuity stimuli were presented for 70 ms followed by the local random dot masks, which were present on the screen until response.

A central white (46.5 cdm^−2^) fixation cross was present throughout, and observers were instructed to maintain fixation on it during the whole experiment (see figure 1). The observers performed a Vernier acuity discrimination task, judging whether the upper line of a pair was shifted to the left or right relative to the lower one (among distractor Vernier acuity stimuli). The upper line of the pair of lines was displaced to the left or right by 6, 12, 18, 30 or 42 arc min. The vertical distance between the two was 0). The Vernier acuity stimuli were presented in white (same brightness as the fixation cross), the length of each line of a pair was 1.3 arc deg and the line thickness was 36 arc min. The local random-dot masks were 2.5° by 2.5° (dot size  = 12 arc min) and the dots were either black (5.67 cdm^−2^), or white. The cue was a black circle (line thickness 36 arc min) surrounding the location of the target. The radius of the cue was 1.7 arc deg. The stimuli were presented on a mid-grey background (21.7 cdm^−2^). The distance of the Vernier acuity stimuli from the fixation point was 13 arc deg.

#### Procedure

A trial (see figure 1) started with the presentation of a central fixation cross, followed 1200 to 1700 ms later (determined randomly) by the cue, presented 50, 100, 150 or 200 ms before target appearance. To increase the number of repetitions of any given CLT, above what would be expected by chance, the probability that the same CLT as presented on the previous trial would be repeated was higher than 0.25. The probability that the CLT would be the same as on the previous trial was equal to 1 - N(0.1 - (0.01N)) where N is the present number of occurrences of the given CLT. Thus, following the first presentation of a given CLT (N = 1), the probability of repetition of that same CLT was .91 and so on (see 2,33 for some applications). This probability function was set to asymptote at the probability 0.75 (when N is  = 5), and when N was >5 the probability of any given CLT was set to 0.25 at N = 6, 7 or 8 (determined randomly). The maximum length of a streak of consecutive trials of the same CLT was thus 8.

The Vernier acuity discrimination stimulus followed at the cued location (presented for 70 ms). The discrimination task involved a judgment of whether the top line of the two in a pair (see figure 1) was displaced to the right or left relative to the lower one. On any given trial, the target appeared randomly in one of the six possible target locations (as indicated by the cue, see figure 1). The other locations contained distractor Vernier acuity stimuli, presented simultaneously to the target, which were irrelevant to the task. Local random-dot masks followed which covered the area where the cue, target and distractors appeared previously. The observers had to indicate the displacement of the upper line relative to the lower one by pressing either 4 (for left displacement) or 6 (for right displacement) on a standard keyboard. Testing was performed in a dimly lit testing room (with no screen reflectance from the ambient light source). The viewing distance was 40 cm. A chin rest was used to ensure that the viewing distance was constant throughout the experiment and to stabilize the observer's head. The observers were instructed not to attempt to move their eyes. Note that they reported that they stopped even attempting moving their eyes following the practice trials since they were never able to move their eyes quickly enough in response to the cue to catch the target before the mask appeared. Before participating the observers were informed about what participation would involve, and were told that they were free to terminate the study at any time. All the observers were experienced observers and had previously given written consent for participation in similar studies, and written consent was thus not deemed necessary for the current experiment. The methods in the study, including the waiver of written consent, were approved by the ethics board of the faculty of Social Sciences at the University of Iceland.

#### Results of experiment 1

We plotted the proportion of rightwards responses as a function of displacement. We also looked at the response times as a function of cue-lead time repetition, but those did not vary as a function of the experimental manipulations. We fitted psychometric functions to the results to measure how discriminable left versus right displacement were for the different number of repetitions of CLT. The following equation was used to fit the psychometric functions:
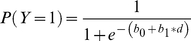
1where *P*(Y = 1) denotes proportion correct, Y is the is the dichotomous response, *b_0_* and *b_1_* are the regression coefficients and *d* denotes the size of the Vernier displacement.

We obtained the slope (the numerical derivative) at displacement  = 0. of the psychometric function for each of the 16 combinations of CLT and cue repetition. Linear fits for the slopes as a function of CLT repetition were estimated, collapsing over CLT, except for observer VK where separate fits were calculated for each CLT, since a separate logistic regression showed a significant 3 way interaction between Vernier displacement, cue-lead time and repetition, which indicates that the effect of repetition was not uniform for the different CLTs. To assess the statistical significance of the results, confidence intervals were calculated for the slope of the linear fit using 1000 bootstrap repetitions (percentile method) [31] except for observer VK where 5000 repetitions and the BCa method was used to calculate confidence intervals for each individual CLT due to a bias in the resampled statistics [32].

**Figure 2:**
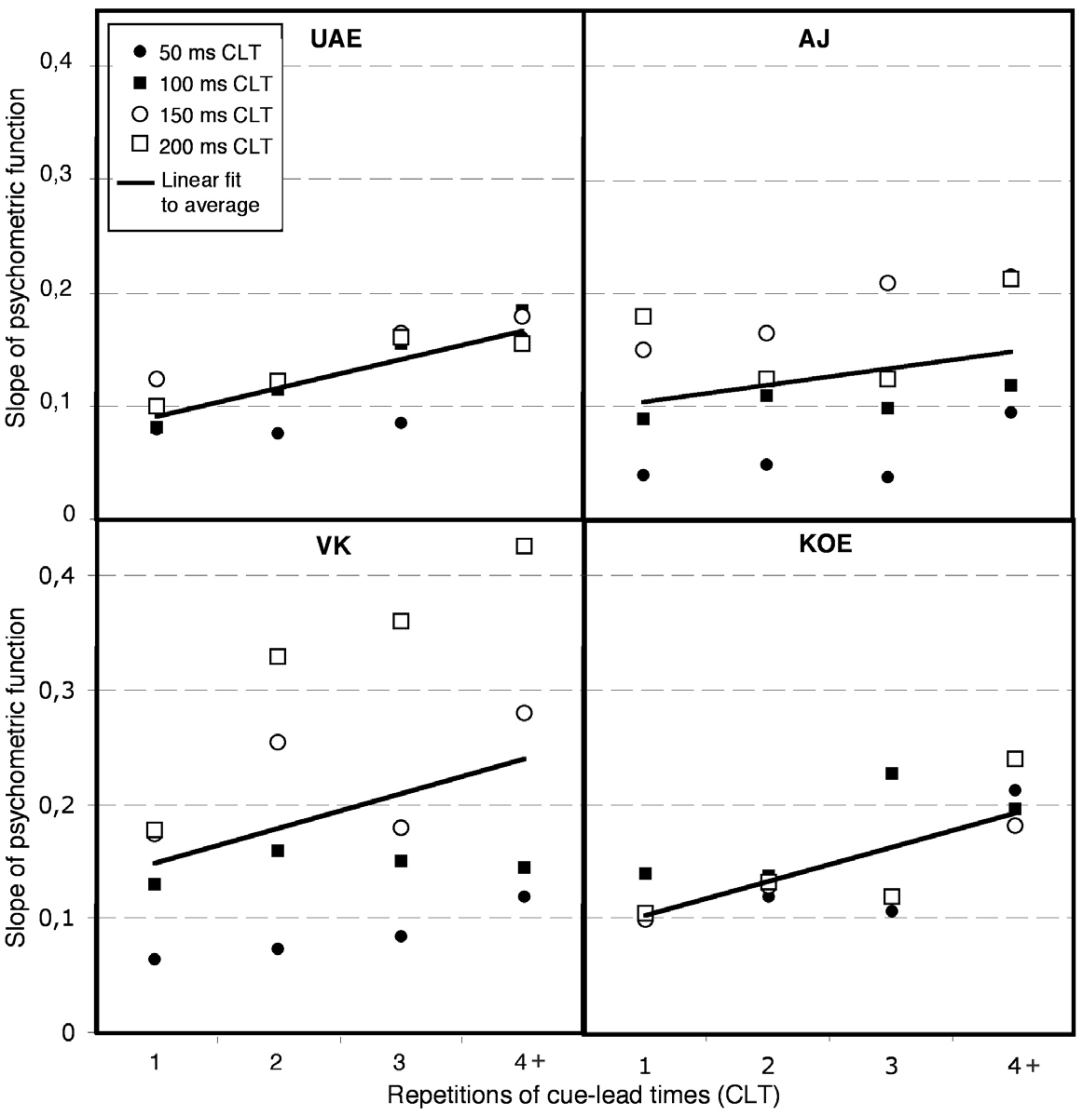
The slopes of the weighted psychometric functions for the 4 observers in experiment 1 as a function of the repetition of CLTs, shown separately for each of the 4 different CLTs (different symbols). The psychometric functions show the proportion of “right” responses as a function of CLT. The solid lines show the best linear fit collapsed over different CLTs.

Figure 2 shows the slopes of the psychometric functions plotting the proportion of *right* responses as a function of displacement size of the Vernier acuity stimulus for the four observers. They are plotted as a function of how often the same CLT was repeated (once, twice, thrice, or four times or higher). Since the slope measures essentially how discriminable leftwards and rightwards displacements on the Vernier target are, we can assess the difficulty level of the task as a function of how often the same CLT was repeated.

The line of best linear fit showed a significant positive slope for observers AJ (0.016, CI: 0.005–0.026), KOE (0.032, CI: 0.020–0.040), and UAE (0.028, CI: 0.020–0.038), while for observer VK the slopes were positive for three of four CLTs (50 ms [0.032, CI: 0.013–0.045], 150 ms [0.024, CI: 0.002–0.067] and 200 ms [0.081, CI: 0.047–0.154] but for the 100 ms CLT the results were inconclusive (−0.002, CI: –0.022–0.013), since the bootstrapped confidence intervals include a slope of zero.

In sum, the slope of the psychometric function increased with CLT repetition for 15 of the 16 cases that we tested with the CLT of 100 ms for observer VK an exception. The significant positive slopes of the linear fits show that this increase in discriminability as the CLT is repeated is statistically significant.

#### Discussion of Experiment 1

The results from experiment 1 clearly indicate that discrimination of whether the upper line on the Vernier acuity target was shifted leftwards versus rightwards became better with between-trial repetition of CLTs. Put another way, the displacement becomes more discriminable with increased number of repetitions of CLT, as the increased slopes of the psychometric function with repetition conclusively show. This indicates that as CLT is repeated, attention shifts become more efficient, most likely reflecting that additional attentional resources are recruited [33]. The attentional system appears to learn *when* to attend to a particular location as the CLT is repeated. The data in figure 2 also show that as the CLTs become longer performance gradually improves, consistent with previous results (13,14,16).

Nakayama, Maljkovic & Kristjánsson ([18], see also [14]) argued for the existence of a primitive memory system for attention deployments. This memory system, they argued, “automatically links separable feature of objects to the act of attentional deployment” ([18], p. 397). We wish to propose that the current results add temporal consistencies to this general scenario, that transient attention shifts are facilitated with repetition of CLTs, not only by repetition of pictorial characteristics of a cue.

### Experiment 2 – Consistency effects upon peripheral cueing, or non-spatial alerting from temporal consistency?

So far we have not disentangled two possible accounts for the results of experiment 1: 1) That consistency can serve as a general non-spatial alerting signal. Westheimer & Ley [34] and Lasley and Cohn [35] observed that orientation discrimination thresholds, stereoacuity thresholds and luminance increment detection are improved when the discrimination targets consistently appear at the same time relative to a warning signal. These are examples of such non-spatial alerting since their targets always appeared in the same location. 2) The second possibility is that the *spatial* precuing effect is enhanced with the between-trial repetition of CLTs.

**Figure 3:**
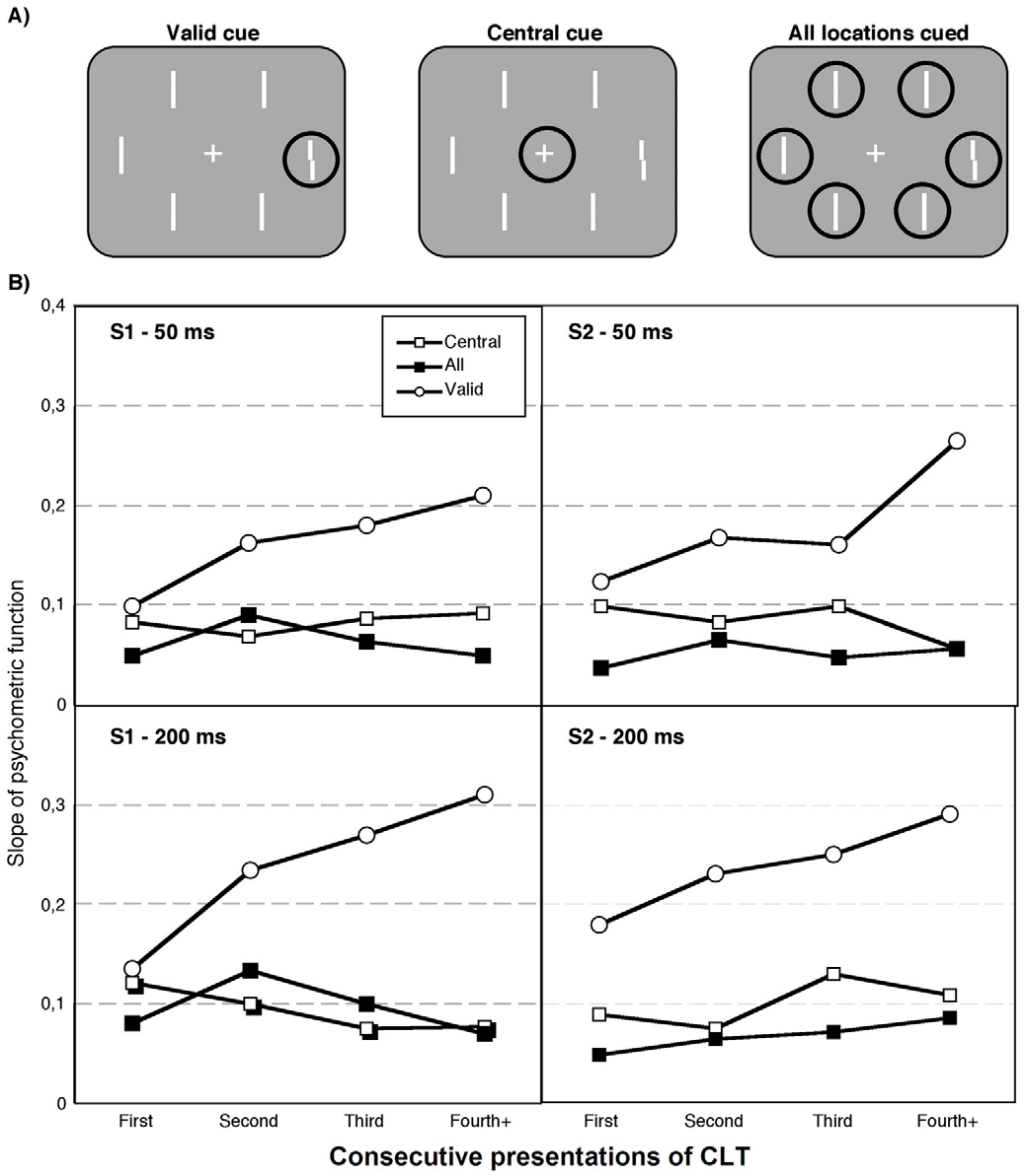
The three different cue types used in experiment 2 along with results. Panel A shows the three cue types used in experiment 2. The slopes of the psychometric functions (see methods for experiment 1) are shown in panel B for the two naïve observers (S1 and S2) for the two different CLTs as a function of trial-by-trial repetitions of CLT. The lines show linear fits to the data for the respective conditions. All the slopes for the valid cue increased significantly with CLT repetition as assessed with bootstrapped confidence intervals, but this was not the case for the “all” and “central” cue conditions.

To disentangle any non-spatial alerting component from spatial precueing effects [16], [26]–[27], [36], we contrasted three types of precue in experiment 2, only one of which was spatially informative (see figure 3a). In the other two cases the precues were non-informative, either similar precues appeared at all locations, or the precue was presented at screen center, around the fixation point (see figure 3A).

Another, albeit related, motivation for experiment 2 was to assess the actual benefits of the precue used in experiment 1. Many studies have indeed shown benefits from spatial precues and have often compared performance to baseline measures such as a neutral non-informative “cue” appearing at the center of the screen, which would certainly be a useful baseline measure here.

## Methods

Three different cue types were used in experiment 2 (see figure 3A), a valid peripheral cue, a neutral cue which appeared around the fixation point at screen center, and in the third condition cues appeared at all six possible target and distractor locations. Two naïve observers took part in 4200 trials each, 1400 with each cue-type. Trials were run in blocks of 100 trials and it was randomly decided which blocks would have cues of which type, with the only constraint that there were 14 blocks for each cue-type.

The upper line on the Vernier acuity targets was displaced by 10, 18, 26 and 34 arc min relative to the lower one. This time the CLTs were determined randomly between trials, and to cut down on the required amount of trials we only used the CLTs of 50 and 200 ms. The distractors were now straight bars to distinguish them from the target when the uninformative cues were used. As before, performance was analyzed by calculating the slopes of psychometric functions (see equation described for experiment 1), separately for each CLT and cue type, and as a function of whether a particular CLT was repeated or not. Otherwise methods were similar to experiment 1.

### Results and discussion of experiment 2

The results of experiment 2 are shown in figure 3B. The slopes of the psychometric function increase with CLT repetition, but importantly only for the valid spatial precue (open circles), not for the two types of spatially neutral cue (the “central” and “all” cues). Any benefit of temporal consistency upon deployments of transient attention seems to require an explicit spatial cue about the targets upcoming location since the discriminability of left versus right on the Vernier target only improves with CLT repetition for the valid cue. This cannot be attributed to a benefit due to non-spatial alerting, since the two different spatially non-informative cues did not lead to any improvements with CLT repetition.

The line of best fit to the slopes of the psychometric function as a function of repeating CLT was positive for all valid cues. The slope and bootstrapped confidence intervals for the valid cues were for S1 (50 ms cue): slope  = 0.21, CI: 0.023–0.46; S1 (200 ms cue): slope  = 0.45, CI: 0.142–0.65; S2 (50 ms cue): slope  = 0.38, CI  = 0.089–0.52; S2 (200 ms cue): slope  = 0.28; CI: 0.049–0.51. A similar analysis for the uninformative cues (“central” or “all”) showed that there was no significant increase in performance as CLTs were repeated – the confidence intervals all included a slope of zero.

The results from experiment 2 also form a baseline comparison to show that the task that we used in experiment 1 does indeed lead to a large effect of validly cueing the upcoming target location, compared with uninformative cues, consistent with previous results [14], [27], [28]. Note that Milliken et al. [37] found a benefit from temporal consistency with uninformative cues, which might seem to contradict the findings here, but in their case there were only two possible target locations and the cue that they used appeared at either location (the cue was not predictive of the target location) which makes a direct comparison between these two studies difficult.

### Experiment 3 – The role of explicit knowledge of cue lead times

One important question with regard to the outcome of the two foregoing experiments is whether observers were able to use voluntary, or endogenous attention [39] to influence their attention shifts according to the task demands, in this case according to the CLT on the preceding trial. Attentional allocation on the timescale investigated here has often been thought of as automatic and reflexive, however. Kristjánsson et al. [13], [14] have shown that this allocation can nevertheless be strategic, based on what has occurred on the preceding trials, albeit in a non-conscious way. In experiment 3 we attempted to assess whether observers are able to use explicit knowledge of the presentation order of the CLTs to facilitate performance on the same Vernier acuity task as was used in experiments 1 and 2.

To test any effects of explicit knowledge upon benefits from repetition of CLTs, we applied a variation upon the methodology introduced by Maljkovic & Nakayama [1] and Kristjánsson & Nakayama [14] to the task and design used in the preceding experiments. Maljkovic & Nakayama ([1], exp. 4) told their observers who searched for a target diamond of odd color relative to distractors that a target of the same color would always appear on two consecutive trials (e.g. red) followed by two presentations of a green target followed by two red targets and so on. In spite of this, the observers showed the distinctive priming pattern in that the response times were reliably faster when the same target color was repeated than when it changed. The observers were not able to willingly “overcome” the priming effects from target color repetition even when they knew about the presentation order.

Similarly, Kristjánsson & Nakayama ([14], exp. 5) using a peripherally cued Vernier acuity task compared two conditions: 1) Where the target would alternate between appearing at the red and green half of the peripheral cue. 2) Where the probability of the target appearing again at the e.g. green location on the peripheral cue was higher than chance. This was independent of the absolute position of the cue and target on the screen (which varied randomly). In other words, in the second case the consistency occurred in object-based coordinates. Importantly, the observers were informed of this before they performed each condition, and were encouraged to use this knowledge to try to improve their performance on the Vernier acuity discrimination task. Kristjánsson et al. found, similarly to Maljkovic and Nakayama before, that observers were unable to use this knowledge to aid their performance on the task, while they still showed the characteristic learning pattern if the target appeared at the same colored part of the cue from one trial to the next.

**Figure 4:**
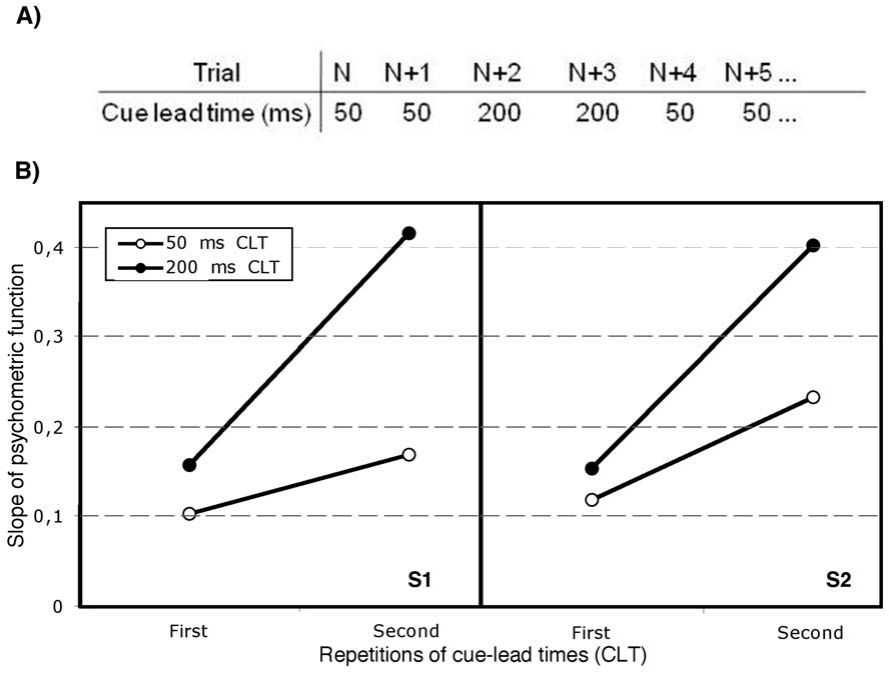
The design and results from experiment 3. Panel A shows the sequence of CLTs on consecutive trials relative to any given trial *N*. Each of the two CLTs was presented twice in a row before switching to the other CLT for two trials, and then back again to the other CLT for two trials, and so on for the 100 trials in each of the 20 trial blocks. Panel B shows the slope of psychometric functions for the two observers for the two cue lead times as a function of whether a particular CLT was repeated (Second) or not (First).

Following this, we informed our observers, that there would be two different CLTs (50 ms and 200 ms, to cover the range of CLTs tested in experiments 1 and 2) and that these would always be presented in pairs of consecutive trials (see figure 4A). The main question of interest was whether performance would be facilitated by observers' knowledge of when, within this short time from the cue presentation the target would appear. In other words, would they be able to allocate their attention at will at particular instances in time, within the time window where transient attention operates, or would they instead show the benefit from repeated CLTs between trials, as we saw in the previous two experiments?

#### Methods

Two naïve observers performed a cued Vernier-acuity discrimination task similar to the one used in experiments 1 and 2. The cue always correctly indicated the location of the upcoming target. The observers were only informed that the CLTs would be 50 ms two times in a row followed by two trials with a CLT of 200 ms, again followed by two trials with a 50 ms cue and so forth (see figure 4A), but otherwise they were naïve with regard to the experimental questions. With this manipulation the observers always knew which CLT would be presented next. Each observer participated in 2000 trials, run in blocks of 100 trials. Performance was analyzed as before by estimating slopes of psychometric functions, separately for each CLT and as a function of whether a particular CLT was repeated or not. The different displacements of the upper line relative to the lower one tested in experiment 3 were 10, 18, 26 and 34 arc min. The distractors were also Vernier displacement stimuli. Methods were otherwise similar to what was presented for the two foregoing experiments.

#### Results and discussion of experiment 3

For each observer we obtained the psychometric slope at zero displacement for each of the four combinations of CLT (50 and 200 ms) and CLT repetition (first and second presentation). Linear fits were found for each CLT and confidence intervals for the slope of each line were estimated using 5000 bootstrap repetitions with the BCa method [32].

The results of experiment 3 are shown in figure 4. Performance was much better for the second presentation with the same CLT and when the CLT changed, performance became worse even though this change was entirely predictable. The slopes of the psychometric function were thus steeper when the CLTs were repeated, than when they changed.

The line of best fit showed positive slopes for both observers at both CLTs. For observer RMK the slope was 0.116 (Bootstrapped CI: 0.04–0.22) for the 50 ms CLT and 0.25 (CI: 0.10–0.44) for the 200 ms CLT. For observer SPD the slope was 0.07 (CI: 0.004–0.13) for the 50 ms CLT and 0.26 (CI: 0.14–0.48) for the 200 ms CLT, showing that the slopes increased significantly in each case with the repetition of CLTs.

The results of experiment 3 show that performance was much better for the second than the first in a pair of trials with the same CLT. When the CLT changed, performance became worse even though this change was *entirely* predictable. The slopes of the psychometric function were significantly steeper when the CLTs were repeated, than when they changed, even if the observers knew about the upcoming CLT. Experiment 3 shows that observers *cannot* use their explicit knowledge of the upcoming CLTs to aid performance on the Vernier acuity discrimination task. They cannot choose *when* they attend to the cued location with such brief CLTs as were used here, but do nevertheless show the improved performance as CLT repeats.

The results of experiment 3 draw another parallel between temporal attending in transient attention as studied here and previously demonstrated learning phenomena in transient attention shifts, in that they show that the learning is not under voluntary control, and occurs in essence in violation of an observers intentions. Finally it should be noted that both observers reported that it was quite impossible to try to apply attention *at will* within these very brief CLTs, similar to what observers in the previously mentioned studies reported.

## Discussion

Our visual systems clearly prefer consistency to uncertainty. Previous results have shown how reflexive shifts of transient attention [14], [17]–[18], [38] or reflexive attentional capture effects [39]–[43] can be modified by spatial [13] and featural [14] consistencies. Such learning has even been found to affect landing points of express saccades [44], perhaps our most reflexive visual behavior [45]–[46]. Between-trial priming has even been found for the size of the attentional focus [47]. Even more surprisingly, Fuggetta et al. [48] observed improved visual search performance when a target appeared at a location which could be expected from principles of good continuation of the target's successive position from one trial to the next.

The current results add another dimension to this scenario, showing for the first time, how temporal consistencies in CLTs, from one trial to the next, lead to improvements in Vernier acuity discrimination performance following deployments of transient attention in response to peripheral cues. This is another surprising example of how such attention shifts, which have often been considered reflexive, can be modified depending on the task demands in each case. Transient attention shows learning of CLTs from one trial to the next as was found previously for position and features. Experiment 2 shows that this benefit from temporal consistency is not simply due to a non-spatial alerting signal stemming from consistency but that the consistency affects the spatial cueing effect itself. Finally, the results from experiment 3 show that this learning is not under any form of voluntary control, which draws another parallel with those previously demonstrated learning effects.

### Temporal uncertainty effects

While effects of spatial and featural consistency upon attentional orienting are well documented, less is known about the effects of uncertainty with regard to shifts of attention in time, while researchers have certainly addressed such questions in the past. An early example comes from the early studies of Woodrow [49] who tested response times following the presentation of auditory signals. When the time from the warning signal to when a response was to be made was variable, this led to performance decrements compared to when this time interval was constant (see also [50]–[51]).

In the literature on visual psychophysics some studies have shown how temporal consistency leads to improvements in performance. If a stimulus appears at a time that the observers expect it to appear at, performance is much better than otherwise. Lasley and Cohn [35] tested sensitivity to luminance increments and found that certainty about when an increment occurred led to better performance than when the increment appeared at an unpredictable time. Westheimer & Ley [34] found that orientation discrimination thresholds were elevated by 20% with uncertainty as to when a target appeared compared to when it consistently appeared at the same time, and for stereoscopic disparity thresholds the increase was as large as 40%. What these results indicate is that observers can have very precise control over when they attend [25], [30].

Note that these effects are more likely to reflect strategic processes on the part of the observer than those under investigation here, since the timescales involved allow voluntary shifts of attention, whereas the longest times between cue presentation and target presentation in the current studies were 200 ms. The current results apply, in other words, to what has been termed *transient* attention [16]. The results from experiment 3 show that observers cannot *willingly* deploy attention at a particular time with such short time intervals between cue and target onset. Our results therefore show that this can occur strategically, but only in an automatic, non-conscious way, consistent with previous results on transient attention [17].

### Relation to previous studies on temporal attention

Previous research has made it clear that attentional allocation can benefit from temporal consistencies. Niemi & Nätäänen [25] reviewed a large amount of evidence that certainly suggests that observers can time their attending accurately, if the foreperiod is predictable between trials. The larger the temporal uncertainty, the slower are the responses to a trigger stimulus. What is new in the current results is that this is shown to build up quickly and then gradually increase from trial to trial and apply to extremely rapid shifts of visual attention (as fast as to attention shifts in response to 50 ms cues).

In the field of visual attention, Lamy [52] found that temporal expectancies could modulate attentional capture. When the time interval between the onset of an irrelevant singleton and a search array was always constant, attentional capture was greatly attenuated, showing that the observers could discount the presentation of this irrelevant singleton provided that it appeared at a predictable point in time. A related result [53] is that explicitly cueing the observers as to the target onset asynchrony between two targets in a dual target rapid serial visual presentation attenuated the attentional blink normally observed under such conditions [54]–[57].

Milliken et al. [37] used a version of the Posner cueing paradigm with *un*informative cues and variable stimulus onset asynchronies (SOA's). For different groups of observers the likelihood of a long SOA cue (900 ms) was highest (66%), 17% for the 500 ms cue and 17% for the 100 ms cue, while for another group these probabilities were reversed. Milliken et al. found that the observers seemed to be able to generate an expectancy for a stimulus within a certain time window depending on these probabilities. Milliken et al. did not have the same sort of breakdown of the within-trial learning pattern as is shown here, but their result clearly shows that exogenous spatial cueing can be modulated by temporal expectancies.

Other evidence shows that we can easily attend to particular instances in time (see also [34]–[35]). Findings of Nobre and colleagues [30] have provided evidence that we can attend selectively in the temporal domain, as we can in the spatial domain – we may choose to orient our attention to a certain instance in time, as we can do with regard to spatial locations. As an example of this, Coull and Nobre ([58], see also [29]) showed that behavioral discrimination performance could be improved if observers were informed with a precue about *when* to attend as has been shown to occur with spatial cues.

### Conclusions

The results reviewed above show that visual and attentional performance can be enhanced with temporal consistencies. The important addition provided by the results presented here is that for the first time this is shown to occur for shifts of transient attention. Experiment 2 shows that this does not reflect a non-spatial alerting signal due to temporal consistency but affects the spatial cueing effect directly. Experiment 3 then shows that conscious control does not play a role in the learning.

The current results add to the evidence for a primitive memory system not under voluntary control [14], [17]–[18], [59], allowing more effective shifts of transient attention to stimuli of importance, in this case allocation of transient attention in time. We wish to propose that this memory system allows us not only to orient (or reorient) our attention more efficiently to an object of current interest, but also more efficiently at the time *when* such an object is most likely to appear, based on our experience of what has occurred on the preceding trials.
